# Environmental enrichment reverses cerebellar impairments caused by prenatal exposure to a synthetic glucocorticoid

**DOI:** 10.3934/Neuroscience.2022018

**Published:** 2022-07-14

**Authors:** Martina Valencia, Odra Santander, Eloísa Torres, Natali Zamora, Fernanda Muñoz, Rodrigo Pascual

**Affiliations:** 1 Laboratorio de Neurociencias. Escuela de Kinesiología. Pontificia Universidad de Valparaíso, Chile; 2 Avenida Universidad #330, Valparaíso., 2340000, Chile

**Keywords:** betamethasone, GR, NT-3, TrkC, anxiety-like behaviours, environmental enrichment

## Abstract

During prenatal life, exposure to synthetic glucocorticoids (SGCs) can alter normal foetal development, resulting in disease later in life. Previously, we have shown alterations in the dendritic cytoarchitecture of Purkinje cells in adolescent rat progeny prenatally exposed to glucocorticoids. However, the molecular mechanisms underlying these alterations remain unclear. A possible molecular candidate whose deregulation may underlie these changes is the glucocorticoid receptor (GR) and neurotrophin 3/ tropomyosin receptor kinase C, neurotrophic complex (NT-3/TrkC), which specifically modulates the development of the neuronal connections in the cerebellar vermis. To date, no evidence has shown that the cerebellar expression levels of this neurotrophic complex are affected by exposure to a synthetic glucocorticoid in utero. Therefore, the first objective of this investigation was to evaluate the expression of GR, NT-3 and TrkC in the cerebellar vermis using immunohistochemistry and western blot techniques by evaluating the progeny during the postnatal stage equivalent to adolescence (postnatal Day 52). Additionally, we evaluated anxiety-like behaviours in progeny using the elevated plus maze and the marble burying test. In addition, an environmental enrichment (EE) can increase the expression of some neurotrophins and has anxiolytic power. Therefore, we wanted to assess whether an EE reversed the long-term alterations induced by prenatal betamethasone exposure. The major findings of this study were as follows: i) prenatal betamethasone (BET) administration decreases GR, NT-3 and TrkC expression in the cerebellar vermis ii) prenatal BET administration decreases GR expression in the cerebellar hemispheres and iii) enhances the anxiety-like behaviours in the same progeny, and iv) exposure to an EE reverses the reduced expression of GR, NT-3 and TrkC in the cerebellar vermis and v) decreases anxiety-like behaviours. In conclusion, an enriched environment applied 18 days post-weaning was able to restabilize GR, NT-3 and TrkC expression levels and reverse anxious behaviours observed in adolescent rats prenatally exposed to betamethasone.

## 1. Introduction

Synthetic glucocorticoid (SGC) administration is considered a type of prenatal stress (PS) because it causes impaired foetal development and vulnerability to different diseases established by foetal programming, which are manifested later in adolescence or adult life [1]–[14]. Moreover, exposure to SGCs in utero during the last quarter of gestation is a pharmacological therapy widely used in the obstetric clinic to accelerate the maturation of lung tissue and subsequently increase the survival of the foetus in cases of premature birth and their use (a single course of betamethasone or dexamethasone) to mature the foetal lung in pregnancies likely to deliver before 34 weeks is recommended [3],[6],[8],[11],[14],[15]–[17].

Longer-term structural changes identified by MRI were observed in the brain of children aged 6–10 years exposed to a single course of antenatal SGCs [16]. Moreover, children born at term and exposed to a single course of betamethasone showed an increased cortisol response to the Trier Social Stress Test at 6–11 years of age [4]. While another similar study described an enhanced stress response at 10 years in children exposed to antenatal betamethasone [18]. Besides, the administration of SGCs increases hyperactivity related to postnatal stress and leads to a higher susceptibility to psychopathology in adolescence and adulthood [1],[4],[11],[15],[16],[19]–[23]. Similarly, in rats and mice, antenatal exposure to SGCs leads to reductions in learning and memory, together with an increase in anxiety-like behaviours in juveniles and adults [24],[25].

SGCs exert their effects mainly through the activation of the glucocorticoid receptor (GR), an intracellular receptor belonging to the family of nuclear receptors that is translocated to the nucleus after binding to cortisol (in humans) or corticosterone (in rodents), where it regulates gene transcription [26],[27]. In both human and rat brains, SGCs target GR, which is very abundant in limbic structures, as well as in the cerebellum [28],[29]. Likewise, the cerebellum has been implicated in several non-motor mental disorders, such as autism spectrum disorder, schizophrenia, and addiction, controlling the reward circuitry and social behavior [30],[31]. In fact, surgical cerebellar resections can change the social behavior, cognition, and emotional responses of the patients [30],[32]. Moreover, the cerebellum is highly vulnerable to exposure to SGCs, causing neuronal degeneration that underlies the cognitive and behavioural side effects that may occur during treatment with SGCs [14],[28],[33]–[35]. However, researchers have not yet determined whether prenatal exposure to SGCs alters the expression of GR in the cerebellum and whether these changes are manifested during postnatal life.

We systematically studied the effect of the prenatal administration of an SGC, betamethasone (BET), on some relevant aspects of neurodevelopment using Sprague-Dawley rats as an animal model [6],[7],[9],[10],[12],[14],[36]. Among the most relevant findings, we found that the prenatal administration of BET alters the dendritic growth of Purkinje cells [7]. However, the molecular mechanisms that underlie these alterations have not been fully described. In this context, neurotrophin 3 (NT-3) and tropomyosin receptor kinase C (TrkC) are neurotrophic complexes that specifically modulate the development of the neuronal connections of the cerebellar vermis [37]–[45]. This complex has been shown to play a crucial role in promoting the maturation and survival of these neuronal cells and is specifically necessary for cerebellar dendritic development [42],[43],[45]–[47]. For example, pioneering studies showed that NT-3 induces the differentiation and migration of granular cells and stimulates dendritic tree development in PCs [37]–[41],[48] and it has been shown that cerebellar granule cells encode expectation of reward [30]. Later, Joo et al. (2014) [43] showed that dendritic morphogenesis and the development of neuronal circuits in the cerebellar vermis require a certain level of NT-3/TrkC signaling. Given the relevance of these neurotrophic factors to specific aspects of cerebellar development and our previously reported evidence, a study of whether antenatal BET administration alters the expression of the NT-3/TrkC neurotrophin complex in the cerebellar vermis of progeny is very important both to elucidate whether this alteration is at least partially responsible for the dendritic deterioration previously observed in BET-treated animals and to determine if this alteration is observed long term in the rat ontogenetic stage equivalent to adolescence.

Importantly, enriched environments (EE) are an experimental paradigm used to study the possible plastic changes induced by an increase in physical exercise, sensory information and social stimulation [49]–[54]. Moreover, changes in brain plasticity associated with EE exposure include increased neurogenesis, cell survival, neuronal connections, dendritic branching and length, and numbers of dendritic spines, and the positive regulation of neuronal growth factors [49],[50],[53],[55]–[57]. Likewise, the restorative effect of an EE on some of the alterations caused by PS has been documented (McCreary & Metz 2016) [50]. For example, we recently reported the effect of an EE on reversing changes in motor function and the expression of synaptic proteins caused by prenatal exposure to BET (Valencia et al., 2019) [36] and that, an EE was able to restore the hippocampal expression of GR in the progeny of rats prenatally stressed through social isolation [49],[51]. However, researchers have not reported whether an EE is capable of regulating the expression of GR in the cerebellum of stressed rats during gestation.

Additionally, the molecular mechanism underlying the effect of environmental enrichment on brain function and anatomy has been partially attributed to the upregulation of proteins involved in neuronal survival and activity-dependent plasticity, such as neurotrophins [54],[55],[58]–[61], which are also involved in the modulation of plasticity changes in the cerebellum [9],[36],[54].

In the present investigation, we evaluated the effects of prenatal exposure to BET on the cerebellar expression of GR, NT-3, TrkC and on anxiety-like behaviors in adolescent offspring and assessed whether exposure to an EE minimized or reversed the possible molecular and behavioral deterioration observed in adolescent offspring prenatally exposed to BET.

## 2. Materials and methods

### 2.1. Animals and experimental design

All procedures were performed according to protocols similar to the standards used in the European Union (Cruelty to Animal Act 1876, Directive for the Protection of Vertebrate Animals used for Experimental and other Scientific Purposes [89/609/EEC]) and were approved by “Pontificia Universidad Católica de Valparaíso” Bioethics Committee. Eight multiparous female Sprague–Dawley rats from the animal vivarium of the Department of Chemistry and Pharmacy at the University of Chile were used in this study. All animals were housed under controlled environmental conditions with a consistent temperature (20 ± 1 °C), a standard day–night cycle (12:12 hours light: dark), and food and water available ad libitum. Once they had mated (two females were housed with one male per cage), the females were placed in individual cages (45 × 25 × 20 cm), and gestational Day 0 (G0) was determined by the presence of sperm detected in vaginal smears.

The pregnant rats were randomly classified into four experimental groups: control (CON) animals, control EE-exposed (CON-EE) animals, betamethasone-treated (BET) animals, and betamethasone-treated and EE-exposed (BET-EE) animals. Pregnant rats were housed in groups of three per cage and were separated into individual cages on the day of delivery. Mothers in the BET and BET-EE groups were administered two subcutaneous injections of BET (0.17 mg·kg^−1^ Cidoten®; Schering-Plough, Inc., Santiago, Chile) in the dorsal neck region, separated by an 8-hour interval, on G20. The CON and CON-EE mothers were administered an equal volume (1 mL) of saline [36]. Behavioural and neuronal assessments were conducted only using male animals to avoid the effect of sex [6],[9],[10],[14],[36]. After weaning (P21), the males were separated (from the mother and the female progeny). Four mothers received BET prenatally, and the other four mothers received saline prenatally. The progeny of 8 mothers were randomly distributed into four groups as follows: 2 progenies whose mother received BET prenatally (BET); 2 progenies who received EE postnatally and whose mother received BET prenatally (BET-EE); 2 progenies whose mother received saline prenatally (CON); and 2 progenies who received EE postnatally and whose mother received saline prenatally (CON-EE). All groups were assessed on postnatal Day 52 (P52), since this day has been considered by several authors and in our previous studies to be equivalent to the period of adolescence [6],[9],[10],[12],[14],[36].

### 2.2. Environmental enrichment

As described by Valencia (2019) [36], on P21, the rats in the CON-EE and BET-EE groups were transferred to an enriched environment once a day for 1 hour for 18 consecutive days (P22-P39). The environment consisted of a large Plexiglas cage (120 × 100 × 70 cm) containing a variety of objects, such as tunnels, shelves, running wheels, ladders, and different manipulatable objects (pieces of wool, paper, cardboard, cotton and other cloth, as well as jars, glass balls and wooden objects), which were changed twice a week to avoid habituation and a new object was added every week. Importantly, all animals in all study groups had free access to food and water during the enriched environment [9],[50],[52],[62],[63].

### 2.3. Behavioural tests

On P52, all groups were assessed for anxiety-like behaviours using two different standardized protocols: EPM and MB tests. In the EPM, each animal was gently placed in the central quadrant of the maze for 5 min (under 300 lux of light with white noise). The number of entries into the open arms of the EPM and the amount of time spent in the open arms were recorded, and the data from the rats that fell from the EPM were discarded. The animals with fewer entries and spent less time in the open arms were considered more anxious [9]. In the MB test, rats were individually placed in laboratory cages (47 × 27 × 15 cm) containing bedding (5 cm deep) and nine marbles (2.3 cm in diameter) arranged in two rows. After a 10 min exposure to the marbles, the rats were removed, and the buried marbles were counted. The bedding was changed between each animal. The marbles were considered buried if at least half of the marble was covered with bedding. More buried marbles indicated increased anxiety [64],[65]. Behaviours in the EPM and MB tests were video recorded and analysed using ANY-maze 4.60 software (Stoelting Co., Wood Dale, USA). All behavioural analyses were conducted between 9:00 am and 3:00 pm, and each apparatus was carefully cleaned with 5% ethanol between each test.

### 2.4. Tissue Extraction

After behavioural evaluations were performed, male animals were initially anaesthetized with a lethal dose of isoflurane. Subsequently, the rats were decapitated, and the brain was quickly removed. Then, the cerebellum was separated from the remaining brain tissue. Twelve cerebellar tissues per group (six for immunohistochemistry and others six for western blot) were randomly selected to perform the immunohistochemical or western blot analyses in the present study. Six cerebellums were used to perform the immunohistochemistry and another six different cerebellums to perform the western blot. Given that the CON-EE group only had 12 males (the lowest number of progenies obtained between the groups), we carried out the maximum number of experiments that was the same for the two indicated procedures and between the study groups. That is, 6N per group for each immunohistochemical and western blot analysis. Regarding the groups whose progenies were greater than 12 males, 6 cerebellar tissues were randomly chosen to be analyzed by immunohistochemistry and another 6 by western blot. The analysis of the immunohistochemical expression of GR was carried out in the cerebellar vermis (cerebellar lobule IX) and in the right and left cerebellar hemispheres. Whereas the analysis of the immunohistochemical expression of NT-3 and TrkC was carried out only in the cerebellar vermis.

### 2.5. Immunohistochemical (IHC) analysis

This analysis was performed by immediately freezing of the corresponding cerebellar vermis in liquid nitrogen. Snap-frozen cerebellar vermis and hemispheres tissues were sectioned (20 µm thick) on a cryostat (Microm HM 525, Thermo Scientific, Microm GMBH, Walldorf, Germany). The Rat Brain in Stereotaxic Coordinates, Sixth Edition of [66], was used to locate the areas in the vermis and in the cerebellar hemispheres. Cerebellar vermis slices were obtained in Lateral 0.18 mm, approximately (Figure 163 from [66]). While the hemisphere cuts were obtained in Bregma −11.64 mm and Interaural −2.64 mm, approximately (Figure 130 from [66]). Sections attached to the slide were washed with PBS twice for 10 min each at 45 rpm and then incubated with 0.5% H_2_O_2_ (Merck, Darmstadt, Germany) for 30 min at room temperature. After two additional washes with PBS (1×), the slices were blocked with 3% BSA (Sigma) and 0.4% Triton X-100 (Sigma) for one hour. The primary antibodies used were GR (G-5): sc-393232 (Santa Cruz Antibodies) (1:100); NT-3 (N-20): sc-547393232 (Santa Cruz Antibodies) (1:500) and TrkC: TA328907 (1:200) (OriGene Antibodies). The tissues were separately incubated with primary antibodies in blocking solution overnight at room temperature. The tissues were then washed three times with PBS (1×) and incubated with biotin-conjugated anti-rabbit (115-065-003; Jackson ImmunoResearch Antibodies biotin-SP-conjugated Affinipure goat anti-rabbit IgG (H + L) (1:500)) and biotin anti-mouse (115-035-003; Jackson ImmunoResearch Antibodies biotin-SP-conjugated Affinipure goat anti-rabbit IgG (H + L) (1:500)) secondary antibodies diluted in 1.5% BSA and 0.2% Triton X-100 for two hours at room temperature without agitation. Then, the tissue was washed again three times with PBS (1×). The avidin–biotin peroxidase complex for antibody detection (VECTASTAIN® Elite ABC kit, Vector Laboratories Inc., Burlingame, USA) was prepared in 1.5% BSA and Triton X-100 for one hour and added to the substrate with diaminobenzidine for 20 min without stirring to visualize the labelled protein (ImmPACT DAB peroxidase substrate, Vector Laboratories Inc., Burlingame, USA). Finally, the sections were washed with distilled water for 10 s. The cerebellar vermis sections were immediately mounted on slides, dried in air, and covered with Entellan (Merck) prior to cover-slipping. Negative controls were obtained by excluding the primary antibody. The cerebellar vermis sections were coded and observed using a Nikon eclipse CI, software NHS elements D 4.60.00 64bits microscope. Immunoreactivity was quantified using ImageJ software (NIH, Bethesda, MD) based on greyscale images (% of controls, arbitrary values). The immunostaining for GR (cerebellar vermis and right and left hemispheres), NT-3 and TrkC proteins in the cerebellar vermis (cerebellar lobule IX) was quantified in 10 images per parasagittal section of tissue (40×) per group. Then, using the ImageJ program (NIH, USA, “white” colour adjusted), images were captured from selected areas of the vermis (cerebellar lobule IX) and of the hemispheres, and the average grey value (sum of the grey values of all pixels divided by the number of pixels) was measured, resulting in a representative optical immunohistochemical staining intensity value for each cerebellar vermis and hemispheres sections. Caution was taken to scan all images under equal lighting conditions. Immunohistochemical analyses were performed by the same investigator who was blinded to the experimental groups of the samples.

### 2.6. Western blot (WB) analysis

Western blot analyses were conducted by homogenizing the corresponding cerebellar vermis (cerebellar lobule IX) and the right and left cerebellar hemispheres in 1× radioimmunoprecipitation assay (RIPA) buffer (50 mM Tris, 0.1% SDS, 1% NP-40, and 0.5% sodium deoxycholate, pH 7.5) containing protease inhibitors (Complete Mini Protease Inhibitor Cocktail, Roche, Mannheim, Germany) with 0.5 mM phenylmethanesulfonylfluoride (PMSF) immediately after harvest. Tissue homogenates were centrifuged at 13,000× g for 10 min at 4 °C. The concentrations of proteins in the homogenates were determined using Qubit® assays. The tissue extracts (20 µg of protein) were electrophoresed on 8% acrylamide gels and transferred to nitrocellulose membranes at 350 mA for 1 hour. The membranes were incubated with blocking solution (5% nonfat milk in 1× PBS-Tween) for 1 hour at room temperature followed by an incubation with primary antibodies against GRs, NT-3 and TrkC in blocking solution overnight at 4 °C. The primary antibodies used were GR (G-5): sc 393232 (Santa Cruz Antibodies) (1:100); NT-3 (N-20): sc-547393232 (Santa Cruz Antibodies) (1:1000); and TrkC: TA328907 (OriGene Antibodies) (1:1000). The anti-beta tubulin antibody ab6046 (Abcam Antibodies) was used as a loading control. The secondary antibodies employed were peroxidase-labelled goat anti-rabbit IgG (H + L) at 1:7000 (115-065-003; Jackson ImmunoResearch Antibodies for NT-3 and TrkC) and peroxidase-labelled goat anti-mouse IgG (H + L) at 1:7000 (115-035-003; Jackson ImmunoResearch Antibodies for GRs) in blocking solution for 1 hour at room temperature. Immunolabelling was detected using a chemiluminescence detection system (WESTAR® Supernova). The densitometric quantification of protein bands was performed using the digital image processing program ImageJ.

### 2.7. Statistical analysis

First, the distribution of the data was determined using the Shapiro-Wilk test (data not shown). If the data exhibited a normal distribution, one-way ANOVA followed by Tukey's multiple comparisons test or two-way ANOVA by Sidak's or Tukey's multiple comparisons test were performed to assess differences in the variables evaluated between the groups. If the data were not normally distributed, the equivalent nonparametric statistical test (Kruskal-Wallis) was used followed by Dunn's multiple comparisons test. GraphPad Prism 9 (GraphPad Software. La Jolla, CA. USA) software was used to analyse behavioral, immunohistochemical and western blot data. The results are presented as the means ± standard errors of the means (SEMs). Differences were considered significant at p < 0.05 in this study. In addition, the effect size was calculated for the variables studied [67].

**Figure 1.**
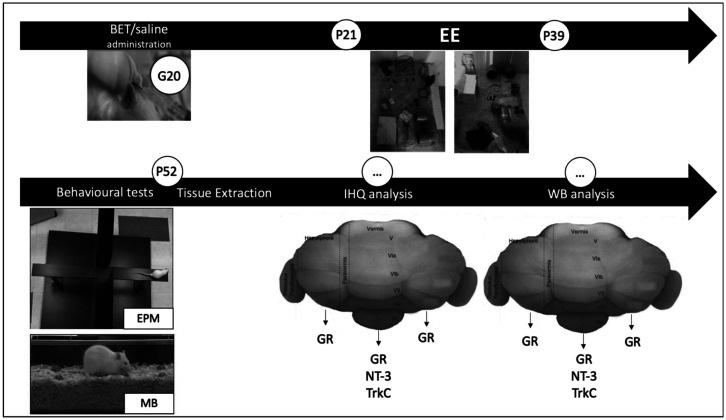
Representative scheme of the methods. BET: Betamethasone; G20: gestational day 20; P21: Postnatal day 21; EE: environmental enrichment; P39: Postnatal day 39; P52: Postnatal day 52; EPM: elevated plus maze; MB: marble burying test; IHQ: immunohistochemistry; WB: western blot; V: cerebellar lobule V; VIa: cerebellar lobule Via; VIb: cerebellar lobule VIb; VII: cerebellar lobule VII; VIII: cerebellar lobule VIII; IX: cerebellar lobule IX; GR: glucocorticoid receptor; NT-3: neurotrophin 3; TrkC: tropomyosin receptor kinase C.

In summary, the experimental desing is schematized in Figure 1.

## 3. Results

### 3.1. Behavioural results

**Figure 2.**
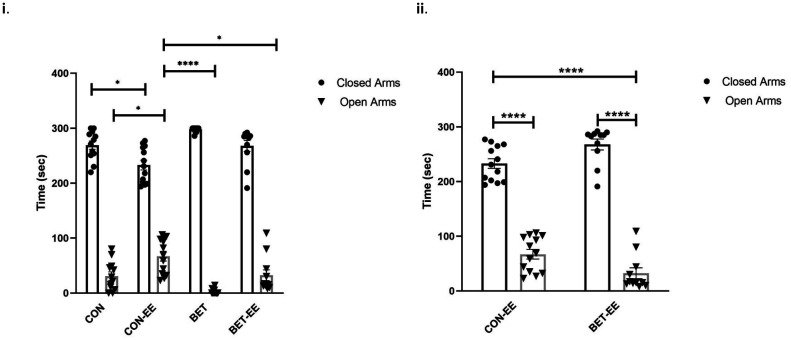
(i) The bar graph show the time (seconds) spent in open arms and the time spent in closed arms from 5-minutes session in the EPM test. The data as presented as the mean ± standard error of the mean from: (CON) control animals; (CON-EE) control- EE treated animals.; (BET) betamethasone treated animals.; (BET-EE) betamethasone-EE treated animals (N = 10; respectivelly) on P52 (age in days). The mean time (sec) in the open arms of the CON, CON-EE, BET, BET-EE groups (23.7, 37.8, 3.2, 25.3, respectively) and the mean time (sec) in the closed arms of the CON, CON-EE, BET, BET-EE groups (275.7, 261.7, 296.6, 274.2, respectively). The data were analyzed using two-way ANOVA (*p < 0.05, ****p < 0.0001) followed by Sidak post hoc comparisons. (ii) The bar graph show the time (seconds) spent in open arms and the time spent in closed arms from 5-minutes session in the EPM test. The data as presented as the mean ± standard error of the mean from: (CON-EE) control- EE treated animals.; (BET-EE) betamethasone-EE treated animals (N = 10; respectively) on P52 (age in days). The mean time (sec) in the open arms of CON-EE, BET-EE groups (37.8, 25.3, respectively) and the mean time (sec) in the closed arms of the CON-EE, BET-EE groups (261.7, 274.2, respectively). The data were analyzed using two-way ANOVA (*p < 0.05, ****p < 0.0001) followed by Sidak post hoc comparisons.

The level of anxiety-like behaviour in all groups studied was assessed using two standardized and widely employed behavioural tests: the EPM (N = 10 animals per group, Figure 1) and the MB test (N = 16, 12, 17, 13, respectively, animals per group). The results were analyzed using two-way ANOVA, followed by Sidak's multiple comparisons test and one-way ANOVA, followed by Bonferroni's multiple comparisons test, respectively. The analysis of the interaction showed significant differences (****p < 0.0001). The behavioural evaluations indicated that BET-treated animals showed significantly more anxiety-like behaviours in the EMP than matched animals in the CON-EE group. As shown in Figure 2, BET-treated animals spent less time in the open arms of the EPM (****p = 0.00067; Figure 2). The multiple comparison between the other groups turned out to be non-significant. The effect size of the EE was 0.912 on the anxiety-like behaviours observed in the animals, which is considered a large effect.

**Figure 3.**
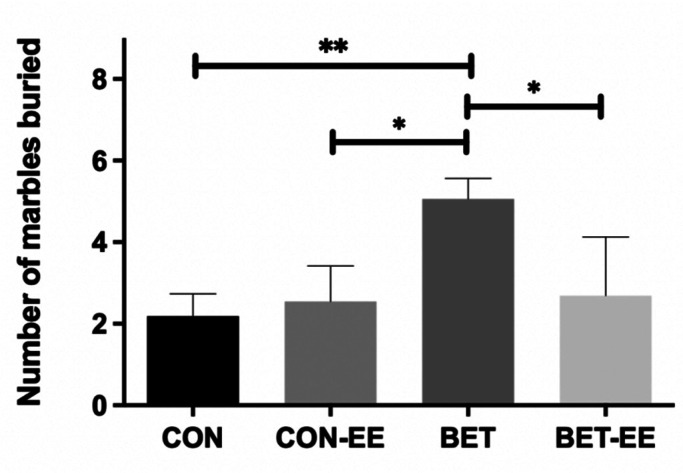
The bar graph shows the total number of buried marbles (10-minute session). The data as presented as the mean ± standard error of the mean from: (CON) control animals; (CON-EE) control- EE treated animals.; (BET) betamethasone treated animals.; (BET-EE) betamethasone-EE treated animals (N = 16, 12, 17, 13; respectively). The data were analyzed using one-way ANOVA (*p < 0.05, **p < 0.01) followed by Bonferroni post hoc comparisons. The statistical bars only show the significant differences among the groups.

Additionally, when analysing the distribution of the MB test results for all groups using the Shapiro-Wilk test, the distribution was parametric (data not shown). The results were analysed using one-way ANOVA, followed by Bonferroni's multiple comparisons test. BET-treated animals buried significantly more marbles than age-matched animals in the CON and BET-EE groups (F(3,53) = 5,9 **p = 0.015; Figure 3). Based on this result, the BET group showed more anxiety-like behaviours than the control group or the group prenatally exposed to betamethasone but also postnatally exposed to an enriched environment.

### 3.2. Immunohistochemistry to detect GR, NT-3 and TrkC protein expression

The immunodetection of each of the abovementioned markers was analysed in the molecular layer (ML) and granular layer (GL) of cerebellar vermis sections from each group. Notably, GR immunostaining was also measured in the cerebellar hemispheres. Values ​​obtained from each group were compared using one-way ANOVA followed by Tukey's multiple comparisons test.

The comparison of the expression of GR in the ML between the groups studied, showed significant differences, the ANOVA value obtained was p = 0.0084; F = 1,241(3,20); size effect = 0.521, medium size effect.

The multiple interactions obtained by Tukey, indicated that compared with matched CON animals, BET-treated animals exhibited significantly reduced GR immunoreactivity in the cerebellar vermal ML (*p < 0.05). The expression of GR in the ML of the cerebellar vermis was lower in the BET group than in the CON-EE group (*p < 0.05). The multiple interactions evaluated between the other groups were found to be non-significant (CON vs. CON-EE; CON vs. BET-EE; CON-EE vs. BET-EE and BET vs. BET-EE).

**Figure 4.**
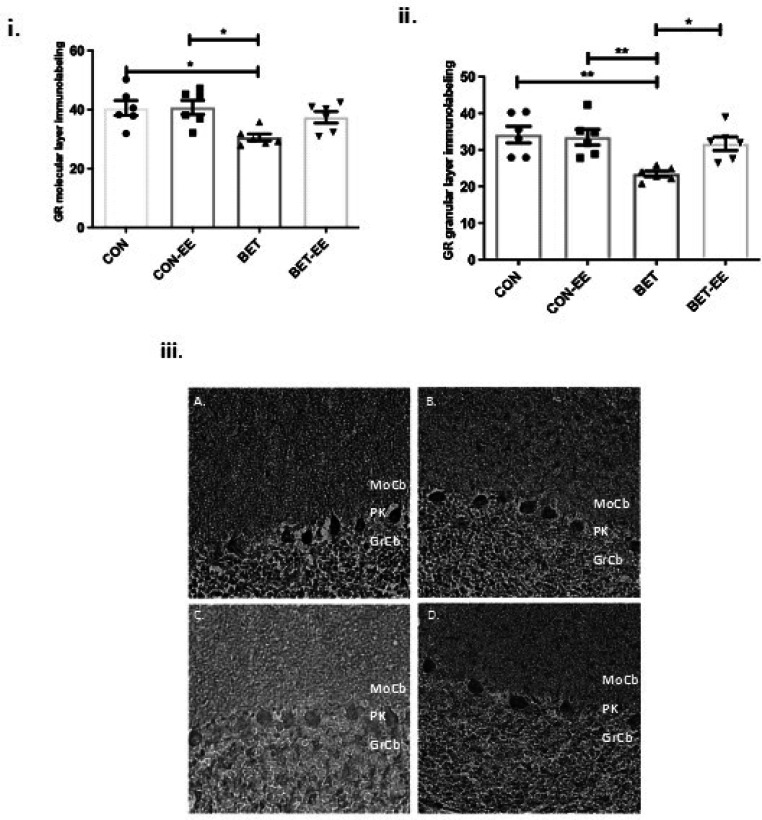
The labelling intensity of GR in the digital photomicrographs was analysed in the cerebellar Molecular Layer (i) and Granular Layer (ii) of the cerebellar vermis of control (CON) animals, the control EE-treated (CON-EE) animals, the betamethasone-treated (BET) animals, and the betamethasone EE-treated (BET-EE) animals on P52. The data are presented as the mean ± standard error (N = 6 per group). The data were analysed using one-way ANOVA (*p < 0.05, **p < 0.01) followed by Tukey post hoc comparisons. (iii) Representatives photomicrographs of Molecular Layer and Granular Layer stained with anti-GR; A: control (CON) animals; B: the control EE-treated (CON-EE) animals; C: the betamethasone-treated (BET) animals; D: the betamethasone- and EE-treated (BET-EE) animals on P52. MoCb: Molecular layer cerebellum; PK: Purkinje cell layer cerebellum; GrCb: Granule cell layer cerebellum.

The comparison of the GR expression in the GL between the groups studied, showed significant differences, the ANOVA value obtained was p = 0.0019; F = 1,714(3,20); size effect = 0.527, medium size effect. The multiple interactions obtained by Tukey, indicated that GR expression was decreased in the GL of the cerebellar vermis in the BET group compared with the CON group (**p < 0.001) and the CON-EE group (**p < 0.001) and the BET-EE group (*p < 0.05). The multiple interactions evaluated between the other groups were found to be non-significant (CON vs. CON-EE; CON vs. BET-EE and CON-EE vs. BET-EE). Figure 4, ii shows representative images of anti-GR staining in the cerebellar molecular and granular layers of the vermis cortex of the animals. GR immunoreactivity was clearly reduced in the cerebellar ML and GL of BET-treated animals compared to that in the cerebellar ML and GL of animals in the other corresponding groups. These results suggest that prenatal exposure to betamethasone decreases GR expression in the cerebellar vermis in the long term, namely, at postnatal Day 52 in male rats.

**Figure 5.**
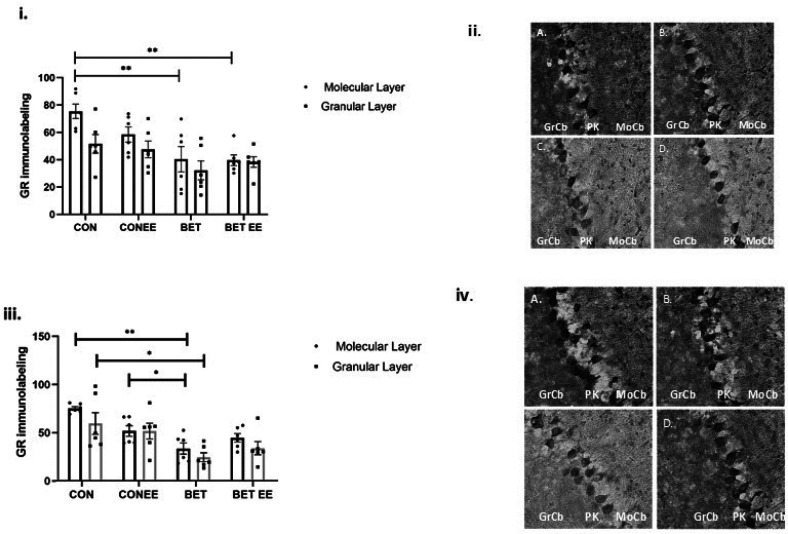
The labelling intensity of GR in the digital photomicrographs was analysed in the cerebellar left hemisphere (i) and the cerebellar right hemisphere (iii) of the control (CON) animals, the control EE-treated (CON-EE) animals, the betamethasone-treated (BET) animals, and the betamethasone EE-treated (BET-EE) animals on P52. The data are presented as the mean ± standard error (N = 6 per group). The data were analysed using two-way ANOVA (*p < 0.05, **p < 0.01) followed by Tukey post hoc comparisons. The statistical bars only show the significant differences among the groups. Representative photomicrographs of Molecular Layer (ML) and Granular Layer (GL) stained with anti-GR of the cerebellar left hemisphere (ii) and the cerebellar right hemisphere (iv); A: control (CON) animals; B: the control EE-treated (CON-EE) animals; C: the betamethasone-treated (BET) animals; D: the betamethasone- and EE-treated (BET-EE) animals on P52. MoCb: Molecular layer cerebellum; PK: Purkinje cell layer cerebellum; GrCb: Granule cell layer cerebellum.

In particular, GR expression was evaluated in the molecular layer and granular layer of both cerebellar hemispheres (Figure 5). In the left hemisphere, a significant decrease in GR expression was observed in the molecular layer of the BET group (**p = 0.0072; Figure 5) and the BET EE group (**p = 0.0055; Figure 5) compared with the control group. In the right hemisphere, a decrease in GR expression was observed in the molecular layer of the group prenatally exposed betamethasone (BET) compared to the CON group (**p = 0.0014; Figure 5) and compared with the CON EE group (*p = 0.0411; Figure 5), it size effect was 0.519 (medium size effect). A decrease in GR expression was also observed in the granular layer of the right hemisphere specifically in the BET group compared to the CON group (*p = 0.018; size effect = 0.241, small size effect). Figures 5 (ii, iv) show representative images of anti-GR staining in the cerebellar molecular and granular layers of the left and right hemispheres from the animals, respectively.

**Figure 6.**
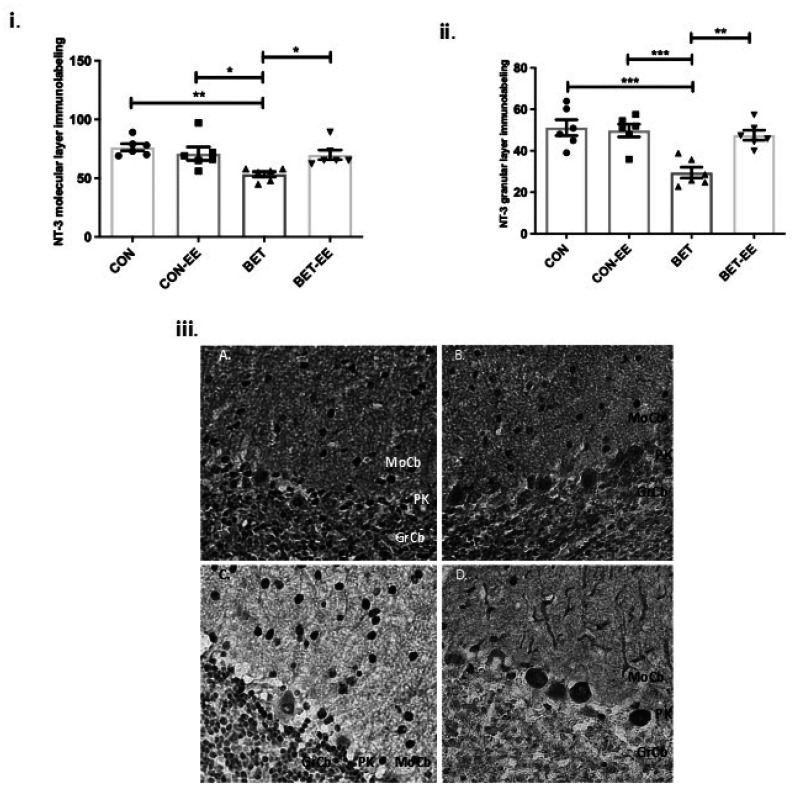
The labelling intensity of NT-3 in the digital photomicrographs was analysed in the cerebellar Molecular Layer (i) and Granular Layer (ii) of the cerebellar vermis of the control (CON) animals, the control EE-treated (CON-EE) animals, the betamethasone-treated (BET) animals, and the betamethasone EE-treated (BET-EE) animals on P52. The data are presented as the mean ± standard error (N = 6 per group). The data were analysed using one-way ANOVA (*p < 0.05, **p < 0.01, ***p < 0.001) followed by Tukey post hoc comparisons. (iii) Representatives photomicrographs of Molecular Layer and Granular Layer stained with anti-NT-3; A: control (CON) animals; B: the control EE-treated (CON-EE) animals; C: the betamethasone-treated (BET) animals; D: the betamethasone- and EE-treated (BET-EE) animals on P52. MoCb: Molecular layer cerebellum; PK: Purkinje cell layer cerebellum; GrCb: Granule cell layer cerebellum.

Importantly, regarding the neurotrophic complex analysed, the comparison of the NT-3 expression in the ML between the groups studied, showed significant differences, the ANOVA value obtained was p = 0.0037; F = 0,3420(3,20); the size effect was 0.587, which is considered a medium effect. In fact, BET-treated animals exhibited significantly reduced NT-3 immunoreactivity in the cerebellar ML compared with that of matched CON animals (**p < 0.01). Furthermore, a significant decrease in NT-3 expression in the cerebellar ML was also observed in the BET group compared to the CON-EE group (*p < 0.05) and the BET-EE group (*p < 0.05). Moreover, a similar change was observed in NT-3 immunoreactivity in the granular layer, the comparison of the expression of NT-3 in the GL between the groups studied, showed significant differences, the ANOVA value obtained was p = 0.0002; F = 0,4175(3,20); the size effect was 0.590, which is considered a medium effect. Compared with matched CON animals, BET-treated animals displayed significantly reduced NT-3 immunoreactivity in the cerebellar GL (***p < 0.001; Figure 6). Additionally, NT-3 immunoreactivity was significantly decreased in the BET group compared with the CON-EE group (***p < 0.001; Figure 6). However, in the granular layer, the BET and BET-EE groups showed differences in NT-3 immunolabelling (**p < 0.01). The multiple interactions evaluated between the other groups were found to be non-significant (CON vs. CON-EE; CON vs. BET-EE and CON-EE vs. BET-EE). Figure 6 (iii) shows representative images of anti-NT-3 staining in the cerebellar vermis cortex of the same adolescent rats.

**Figure 7.**
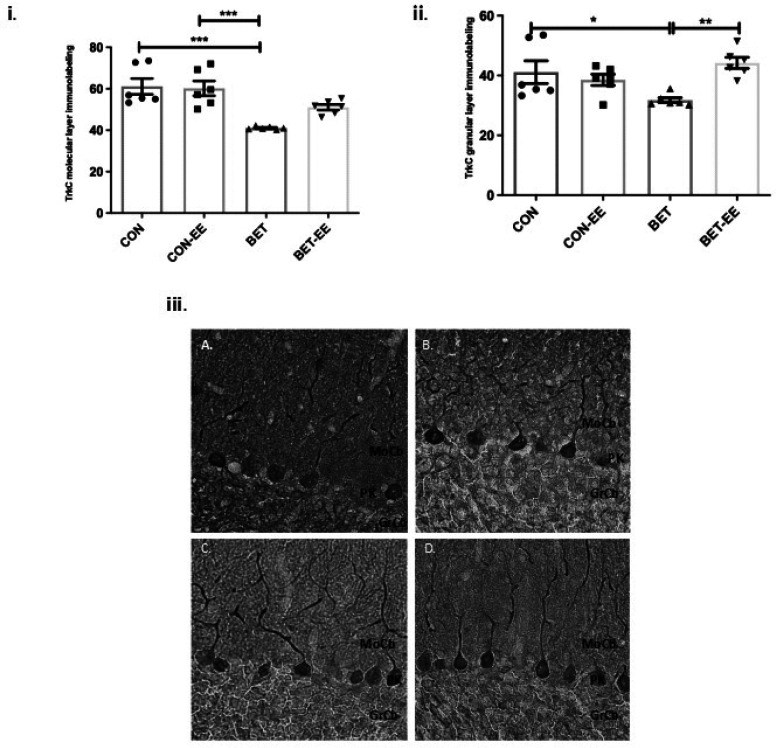
The labelling intensity of TrkC in the digital photomicrographs was analysed in the cerebellar Molecular Layer (i) and Granular Layer (ii) of the cerebellar vermis of the control (CON) animals, the control EE-treated (CON-EE) animals, the betamethasone-treated (BET) animals, and the betamethasone EE-treated (BET-EE) animals on P52. The data are presented as the mean ± standard error (N = 6 per group). The data were analysed using one-way ANOVA (*p < 0.05, **p < 0.01, ***p < 0.001) followed by Tukey post hoc comparisons. (iii) Representatives photomicrographs of Molecular Layer and Granular Layer stained with anti-TrkC (iii); A: control (CON) animals; B: the control EE-treated (CON-EE) animals; C: the betamethasone-treated (BET) animals; D: the betamethasone- and EE-treated (BET-EE) animals on P52. MoCb: Molecular layer cerebellum; PK: Purkinje cell layer cerebellum; GrCb: Granule cell layer cerebellum.

Consequently, the comparison of the TrkC expression in the ML between the groups studied, showed significant differences, the ANOVA value obtained was p = 0.0001; F = 2,321 (3,20); and the size effect was 0.503, which is considered a medium effect. In fact, BET-treated animals exhibited significantly reduced TrkC immunoreactivity in the cerebellar ML compared with that in matched CON animals (***p < 0.001; Figure 7, i) and CON-EE animals (***p < 0.001). The multiple interactions evaluated between the other groups were found to be non-significant (CON vs. CON-EE; CON vs. BET-EE; CON-EE vs. BET-EE and BET vs. BET-EE).

Additionally, when analysing TrkC immunoreactivity in the granular layer, a similar situation was observed. In fact, the comparison of the TrkC expression in the GL between the groups studied, showed significant differences, the ANOVA value obtained was p = 0.0089; F = 1,374 (3,20); and the size effect was 0.486, which is considered a medium effect. For example, anti-TrkC labelling in the BET group was sparser than that in the control group (*p < 0.05) or the BET-EE group (**p < 0.01; Figure 7, i). The multiple interactions evaluated between the other groups were found to be non-significant (CON vs. CON-EE; CON vs. BET-EE; CON-EE vs. BET; CON-EE vs. BET-EE). Similarly, these differences in anti-TrkC immunoreactivity are shown in the representative images presented in Figure 7 (iii).

### 3.3. Western blot analysis of levels of the GR, NT-3 and TrkC proteins in the cerebellar vermis

The expression levels of each protein in the cerebellar vermis and GR levels in the left and right cerebellar hemispheres were measured using western blot analysis. The four groups (N = 6 animals per group) were analyzed using western blotting to determine GR, NT-3, and TrkC expression levels in the cerebellar vermis and GR expression in the left and right cerebellar hemispheres. Bands for the GR, NT-3, and TrkC proteins (approximately 95, 38, and 94 kDa in size, respectively) were observed and quantified using densitometry. The levels of each of the proteins described above were normalized to levels of the total protein loading control β-tubulin. The levels of each of the molecular markers described above in the control group were set to 100%, and the levels in all the other groups were calculated as a percentage of this expression level.

As in the previous analyses, the data obtained were subjected to the Shapiro-Wilk normality test. The distributions of the data obtained from western blotting for all the analyzed markers were not normally distributed (data not shown); thus, the subsequent statistical analyses were performed with the Kruskal-Wallis test, followed by comparisons between the analyzed groups using Dunn's test.

**Figure 8.**
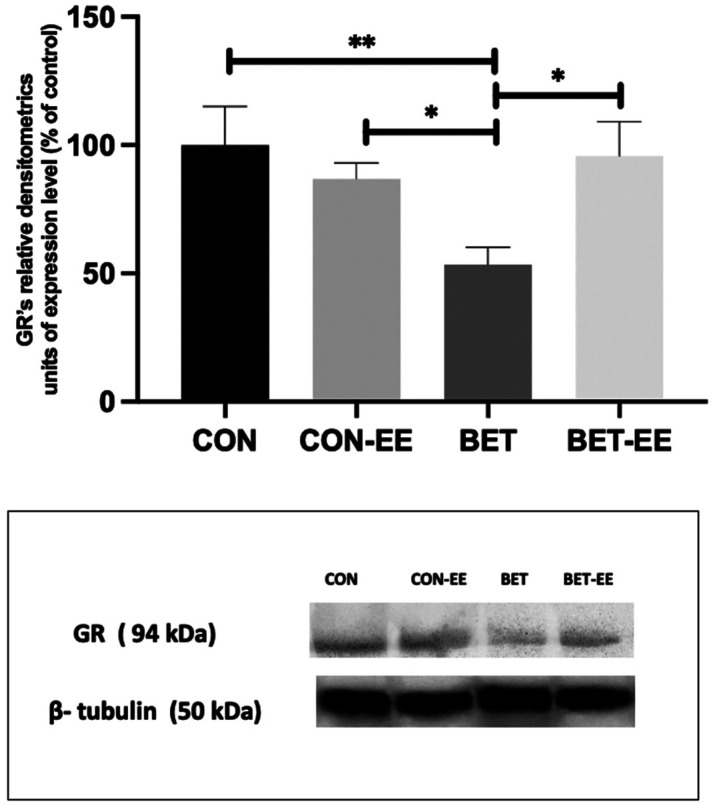
The GR expression levels in the cerebellar vermis of rats prenatally exposed to BET were determined by WB analysis. The data are presented as the mean ± standard error of the control of (CON) animals, the control EE-treated (CON-EE) animals, the betamethasone-treated (BET) animals, and the betamethasone- and EE-treated (BET-EE) animals (N = 6 per group). The data were analysed using Kruskall-Wallis (*p < 0.05, **p < 0.01) followed by Dunns post hoc comparisons. The statistical bars only show the significant differences among the groups.

In this context, BET-treated animals displayed significantly lower GR expression than animals in the CON (**p < 0.001), CON-EE (*p < 0.05), and BET-EE (*p < 0.05) groups (K = 13.46, **p = 0.0037, Figure 8). Likewise, when evaluating the expression of GR in the left hemisphere using western blotting, decreased expression of this protein was observed in the BET group compared to the CON (**p = 0.0010) and CON-EE (*p = 0.0115) groups.

**Figure 9.**
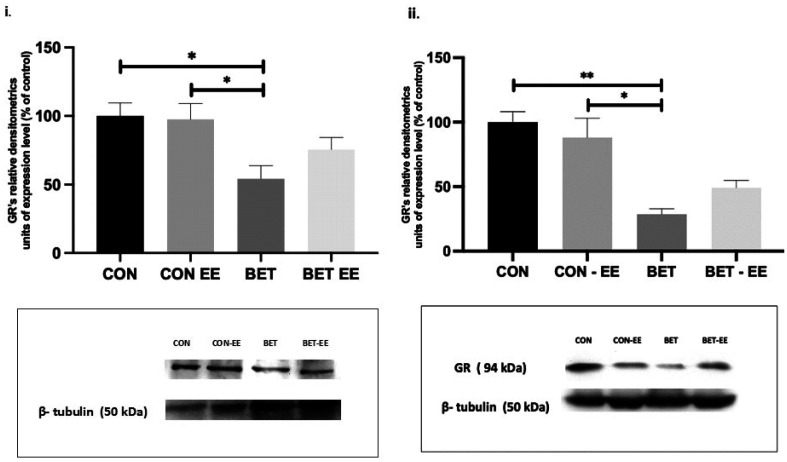
The GR expression levels in the cerebellar right (i) and left (ii) hemispheres of rats prenatally exposed to BET were determined by WB analysis. The data are presented as the mean ± standard error of the control of (CON) animals, the control EE-treated (CON-EE) animals, the betamethasone-treated (BET) animals, and the betamethasone- and EE-treated (BET-EE) animals (N = 6 per group). The data were analysed using Kruskall-Wallis (*p < 0.05, **p < 0.01) followed by Dunns post hoc comparisons. The statistical bars only show the significant differences among the groups.

Similarly, in the right hemisphere, differences in expression were observed between the same groups. Specifically, GR expression was decreased in the BET group compared to the CON (*p = 0.0161) and CON-EE (*p = 0.0290) groups. The graph showing the data is accompanied by representative images (Figure 9).

**Figure 10.**
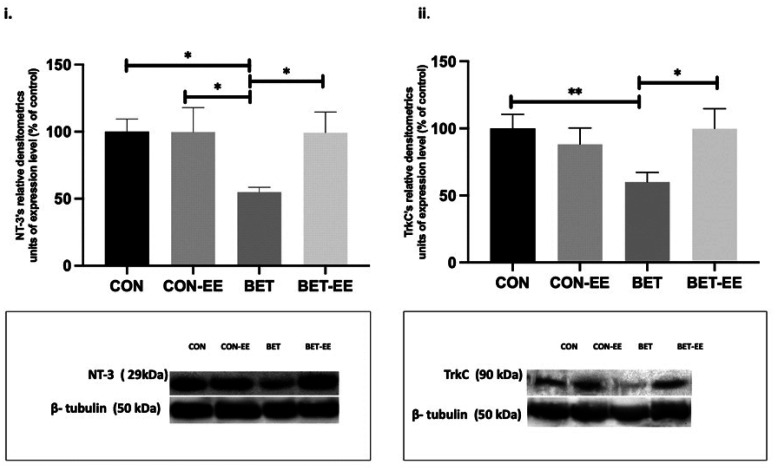
The NT-3 (i) and the TrkC (ii) expression levels in the cerebellar vermis of rats prenatally exposed to BET were determined by WB analysis. The data are presented as the mean ± standard error of the control of (CON) animals, the control EE-treated (CON-EE) animals, the betamethasone-treated (BET) animals, and the betamethasone- and EE-treated (BET-EE) animals (N = 6 per group). The data were analysed using Kruskall-Wallis (*p < 0.05, **p < 0.01) followed by Dunns post hoc comparisons. The statistical bars only show the significant differences among the groups.

Regarding the NT-3/TrkC complex, NT-3 was expressed at significantly lower levels in BET-treated animals than in matched animals from the CON (*p = 0.0123), CON-EE (*p = 0.0329) and BET-EE (*p = 0.0309) group. Finally, in the BET group, TrkC was expressed at a lower level than that in the CON group (**p = 0.0061) and the BET-EE group (*p = 0.0131). The graphs showing the data are accompanied by the corresponding representative images in Figure 10.

## 4. Discussion

We showed that progeny exposed to BET prenatally showed an increase in anxiety-like behaviors, as evaluated using the EPM and the MB test. Rats in the BET group stayed in the enclosed arms of the EPM longer (compared to the CON EE group) and hid more marbles in the MB test than the control group. These results are consistent with previously reported data [17],[21] showing the effect of antenatal exposure to dexamethasone and corticosterone on anxiety-like behaviors in progeny, showing their long-term anxiogenic effects, as has also been shown in our previous studies [9],[12].

The changes in GR expression level observed in the BET group are consistent with previous studies showing a decrease in the level of GR expression in the limbic structures of offspring subjected to antenatal SGCs exposure [13],[68]. The foetal HPA axis is particularly susceptible to long-term programming by glucocorticoids and its dysfunction has been associated with impairment in cerebellar development [69]. These changes are associated with the DNA methylation of a neuron-specific exon 17 promoter of GR (Nr3c1) [20],[70],[71]. One interpretation for this finding is that the GR protein, which is encoded by the NR3C1 gene, binds to cortisol and other glucocorticoids to create a negative feedback loop within the HPA axis that regulates the body's neuroendocrine response to stress [23]. In this regard, the excess methylation of a promoter sequence within NR3C1 attenuates GR expression [20],[70]. Likewise, altered GR mRNA expression due to prenatal stress causes long-term increases in HPA axis reactivity and anxiety-like behaviours [72].

Considering the determinant regulatory role of NT-3 in cerebellar tissue, we suggest that a decrease in NT-3 expression might be involved in alterations in the differentiation and elaboration of the dendritic trees of PCs described in our previous studies [7],[9],[12], since these cells require appropriate levels of NT-3 for normal development [48],[73]. In fact, the addition of exogenous NT-3 alters the morphology of neurites of this cell type [42],[43],[73] and notably, granule cells convey information about the expectation of reward [30],[74]. According to the literature, NT-3 is described as a regulator of synaptic plasticity; therefore, we infer that a decrease in NT-3 expression in an area where PCs and granular cells are located might alter this important process [43]. Similarly, TrkC has also been strongly implicated in neuronal development in the cerebellum. For instance, a study that evaluated mice expressing mutant TrkC showed defects in the morphogenesis of PCs instead of a delay in the development of these cells, suggesting that TrkC is autonomously required for cell growth and dendritic branching in these cerebellar cells [43]. Significantly, functions such as the regulation of the apoptosis of granular cells [73], mediation of synaptogenesis, and neuronal activity are attributed to the NT-3/TrkC neurotrophic complex, in addition to its role in modulating the dendritic arborization of different cerebellar neuronal types [43] (Joo et al., 2014). Accordingly, Joo et al. (2014) [43] show that NT-3 present in presynaptic neurons (granular cells) is necessary for TrkC-dependent competitive dendritic morphogenesis in postsynaptic neurons (PCs), which was previously an unknown mechanism of neural circuit development in the vermis. The functions of the NT-3/TrkC complex are particularly important for the neurodevelopment of granular cells and PCs. Because we have shown that its expression is affected by exposure to BET, we suggest that a decrease in the expression of NT-3/TrkC in the cerebellar vermis might at least partially mediate the decrease in dendritic arborization that we previously observed in PCs and the alterations in functional aspects associated with NT-3/TrkC.

On the other hand, we wanted to evaluate whether an EE was able to reverse the alterations described above. Regarding the effect of EE on anxiety-like behaviours observed in the group exposed to prenatal stress, the BET-EE group hid fewer marbles than the BET group in the MB test. These results are consistent with several studies describing the anxiolytic effect of an EE. For example, EE exposure reduces anxiety-like behaviours and prevents cognitive impairment elicited by acute and chronic stressors [50]–[52],[72],[75]–[77]. Similarly, Leger et al. (2015) [77] found that three weeks of exposure to an enriched environment exerted an anxiolytic effect (measured using the EPM) but also reduced locomotor activity. Therefore, future studies investigating whether this parameter is also altered in our model using the open field test would be interesting.

Regarding the restorative effect of an EE on GR expression observed in the present study, Ashokan et al. (2018) [57] also showed that an EE renormalizes GR signaling within the hippocampal formation along with a reduction in glucocorticoid ligand levels, which is consistent with our data. Likewise, prenatal stress promotes a rapid increase in the nuclear translocation of GR through changes in the transcription factor cAMP response element-binding protein (CREB) activity in the basolateral amygdala [75]. Since an EE regulates CREB activity, this translocation has been postulated as the mechanism by which an EE reverses the deleterious effects of changes in GR expression following exposure to SGCs and the molecular mechanism by which an EE prevents anxiety-like behavior [76]. Thus, we propose that these molecular changes also occur in the cerebellar vermis, which may at least partially explain how an EE reverses the abovementioned alterations in the BET group. Specifically, some biophysical parameters of the granular cells change [78], indicating that the cerebellar circuits together with the functions controlled by the cerebellum are activated by the EE [58].

In addition, EE exposure has been suggested to be a key factor promoting NT-3 expression [55],[59],[61]. According to the results of the present study, an EE restored cerebellar NT-3 expression levels that had been previously decreased by exposure to BET, consistent with a recent study performed by Mc.Creary et al. (2016) [49], who showed that an EE mitigates changes in the endocrine and neuronal markers of stress and increases the expression of hippocampal NT-3 in stressed lineages. Our findings are also consistent with the results reported by Hu et al. (2013) [55], who observed an increase in NT-3 expression mediated by EE exposure in a genetic model of Alzheimer's disease. In this context, exposure to an EE increases the phosphorylation of CREB by activating signaling pathways mediated by the activity of the neurotrophic factors investigated in this work (and their receptors, especially those in the Trk family), and activated CREB subsequently regulates the transcription of several genes involved in the survival of neuronal cells, neuroprotection, synaptic plasticity, differentiation and dendritic spine formation [51],[54],[55],[60],[63].We postulate that an EE is likely to affect CREB activation through the ERK, PI3K and p38 MAPK pathways [54],[55],[60]. Thus, the EE re-establishes the levels of NTF3 and the NT-3 mRNA, as shown by the study by Paban et al. (2011) [59], in which IgG-saporin-immunolesioned rats showed restored NTF3 expression in the cholinergic basal forebrain after exposure to an EE.

Finally, we have shown that short-term exposure (18 days) to an EE restores all the alterations associated with BET exposure described in this study, consistent with our most recent study showing that the same EE restored the expression of various synaptic cerebellar proteins and reversed locomotor problems caused by prenatal exposure to BET [36]. This finding is consistent with the results reported by Murueta-Goyena et al. (2018) [52], who showed that 18 days of EE exposure in early adulthood reduced anxiety-like behavior and by McCreary et al. (2016) [49], who demonstrated that a short environmental enrichment as a powerful intervention for adverse brain programming, mainly in the NT-3 pathway. Therefore, our EE may serve as a powerful intervention to restore cerebellar impairments (mentioned above) caused by prenatal exposure to a synthetic glucocorticoid.

## 5. Conclusions

In this study we observed that the exposure to prenatal BET (i) increased the anxiety-like behaviors (ii) decreased the expression of GR, NT-3 and TrkC, in the cerebellar vermis; (iii) decreased the expression of GR in both cerebellar hemispheres, and that the EE provided in the present study minimized all the previously mentioned alterations. Finally, the effect of prenatal stress on the cerebellum, and the effect of EE to reduce these alterations should continue to be studied.
